# Impact of Pesticide Exposure among Rural and Urban Female Population. An Overview

**DOI:** 10.3390/ijerph18189907

**Published:** 2021-09-20

**Authors:** Bouchra Dahiri, José Martín-Reina, Pilar Carbonero-Aguilar, José Raúl Aguilera-Velázquez, Juan Bautista, Isabel Moreno

**Affiliations:** 1 Area of Toxicology, Department of Nutrition and Bromatology, Toxicology and Legal Medicine, Faculty of Pharmacy, University of Sevilla, 41012 Sevilla, Spain; boudahkha@gmail.com (B.D.); josemartin@eu-salud.com (J.M.-R.); imoreno@us.es (I.M.); 2 Department of Biochemistry and Molecular Biology, Faculty of Pharmacy, University of Sevilla, 41012 Sevilla, Spain; jraguilera@us.es (J.R.A.-V.); jdbaut@us.es (J.B.)

**Keywords:** women, children, pesticides, health, pregnancy, lactation

## Abstract

Pesticides are substances that have become widely used in agriculture and the human exposure to these substances may cause adverse health outcomes. Non-occupational exposure to them can come from many sources, such as food or water. For occupational exposure, many studies have been conducted in men, as they have been mostly in charge of work related to these substances. Nonetheless, the information available concerning the exposure in women is very scarce. In addition, an important differentiation between rural and urban areas has been established, rural areas being known as the most exposed ones due to plantation fields. However, the application of higher concentrations of herbicides in small urban areas is taking a lot of importance currently as well. Regardless of gender, the conditions of exposure, and the environment, the exposure to these pesticides can have different effects on health from early life stages, resulting in different outcomes ranging from neurodevelopmental effects in newborns to different types of cancers. In this review, we discussed the toxicity of the most commonly used pesticides and the main impact on the health of the general population, focusing mainly on the effect in women from both rural and urban areas, and the different stages of development, from pregnancy or lactation to the outcomes of these exposures for their children.

## 1. Introduction

Pesticides are a type of intrinsically toxic substances that are widely used in agriculture to protect crops from pests, diseases, and weeds [1]. According to the Food and Agriculture Organization of the United Nations (FAO, Rome, Italy), a pesticide is defined as “any substance, or mixture of substances of chemical or biological ingredients intended for repelling, destroying or controlling any pest, or regulating plant growth” [2]. Exposure to pesticides can come from many routes, from non-occupational exposure such as domestic applications or through residues in food and drinking water, to occupational settings due to their extended use in agriculture; respiratory (through inhalation), oral and dermal are the main three sources of exposure [3]. Occupational exposure has been mostly studied in farmworkers and its association with diseases such as cancer, neurological pathologies, and the effects on fertility and pregnancy [1,3]. For non-occupational exposure, these residues in food come from dietary intake, especially in products such as fish, meat, and dairy, as they present high lipid fractions with lipophilic compounds that can accumulate and magnify their concentration [4]. Rural areas have been of great concern due to their proximity to the application site. People residing in those areas have been defined as “residents” or the “persons who live, work or attend a school near crop fields treated with pesticides and whose presence is unrelated to work involving pesticides, but whose position might lead them to be exposed” in the scientific literature and by the European Food Safety Authority (EFSA, Parma, Italy) [5]. However, several types of pesticides, especially herbicides, have been applied at higher concentrations in small urban areas like gardens and parks. The indiscriminate application of these pesticides in lawns in schools, parks, and homes, results in the exposure to these chemicals that can vaporize, drifting and rubbing off grass and on the soil [6].

All these chemicals, once applied, affect not only different pests, but also may have certain risks to humans, animals, and the environment, due to their intrinsic properties and associated use patterns.

## 2. Materials and Methods

### 2.1. Search Strategy

All the information was obtained from the Pubmed and Scopus databases, and different published articles related to the objectives of the review were found. All access to these articles was granted trough the library of the University of Seville, which has an agreement with the main editorials to allow free access to full text manuscripts.

The search terms employed in combination with pesticides were: AND health effects, AND health effects AND women, AND health effect AND women AND children. The search criteria were that these terms were in the title, keywords, or abstract. Studies given preference for inclusion were large-scale accidental/occupational exposures or epidemiological studies, prospective or retrospective cross-sectional and cohort studies.

First, a compilation of information about pesticides and health effects was carried out. This process was performed by using pesticides and health effects as keywords in Scopus, where different relevant studies and reviews were found. To find more information about health effect in women, a new search took place in both Scopus and Pubmed using these keywords: pesticides, health effect and women, resulting in the finding of several articles in this regard. Furthermore, the term “children” was added as a new word to health effects, resulting in health effects in children. The number of articles found after all these searches is shown in Table 1.

When searching related topics (pesticides and health effect, pesticides, health effect and women and finally pesticides, health effects, women, and children), relevant information about all these relationships with pesticides and health effects in both women and children was obtained.

### 2.2. Data Collection and Analyses

**Figure 1:**
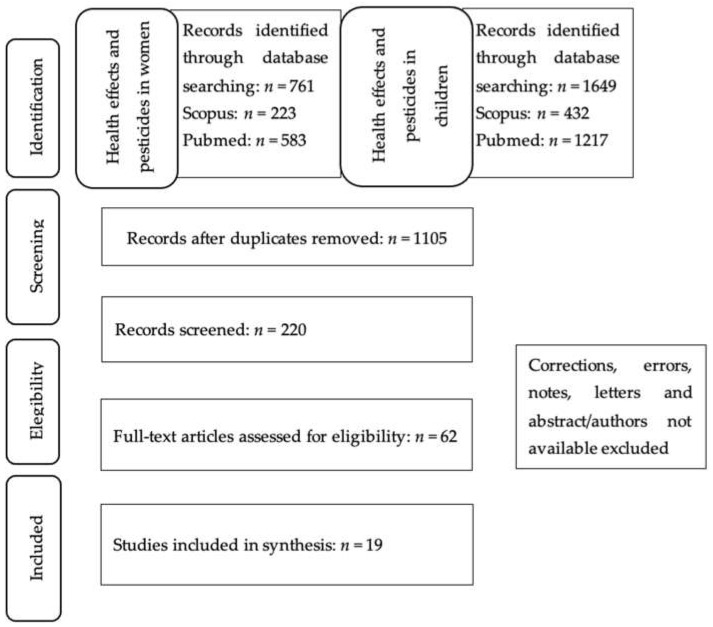
Schematic representation of data handling.

Finally, a compilation of relevant information about the relationship between different pesticides and health effects in both women and children was performed, in all kinds of areas, making a special differentiation between urban and rural areas. In addition, reproductive health, pregnancy, and lactation were taken into account. Data collection was mainly focused on studies that include those pesticides that are more commonly used in agricultural (organophosphates and carbamates) and home environments (pyrethroids) and on the most used herbicide in agriculture and urban gardening, which is glyphosate (GLY), to measure real exposure, and not just the exposure of rural populations or those occupationally exposed. The papers selected for inclusion in this work were based on the title and the screening of both the abstract and full text, if appropriate (Figure 1). Further relevant studies were identified through cross-referencing and author searches.

In order to organize all the information obtained from the selected papers, two databases were built to enter the relevant information in a standardized survey including the items of interest. The first form included these five sections: (1) location of the study; (2) population; (3) type of pesticide studied; (4) health effect on the population of study; and (5) reference. The second form also included the other five: (1) population and location of study; (2) type of sample collected; (3) type of pesticide studied; (4) analytical method used; (5) levels of pesticides detected; and (6) reference.

## 3. Results and Discussion

As a result of our search, 19 studies were obtained that finally were included in the present review. Out of the databases built, one of them focused on the health effects caused by specific pesticides in a specific group of the population that included groups made by both men and women; only women; or women and their children. For the other one, in which mainly analytical records were included, levels of pesticides detected in the population groups were established, together with the methods used for their determination. Finally, a wide variety of results was obtained, all of them establishing that pesticide exposure may occur in different circumstances from occupational to non-occupational sources, not only affecting rural population but also the urban one, with special interest in vulnerable populations such as pregnant women or children being breast-fed.

### 3.1. Toxicity of Pesticides

The toxic effects of pesticides depend on their individual chemical category, the dose and the duration of exposure, as well as the exposure route [3]. The classification of pesticides is a difficult task from the point of view of the health sector. One practical, even less detailed classification is by their target plague (see Table 2). Their classification according to their toxicity is also difficult because there are multiple mechanisms of action described in different organs. The mode of action of pesticides is diverse and sometimes they are not well-defined [7]. In this sense, organophosphates are characterized by their irreversible binding with the enzyme acetylcholinesterase (AChE), which leads to the inhibition of the synaptic transmission [8]. They are also involved in the production of free radicals that could cause some pathophysiology and/or chronic diseases in humans through oxidative damage of lipids, carbohydrates, proteins and nucleic acids, including changes to the DNA structure [9]. Organophosphates can also induce endocrine disruption through a multitargeted process at the hypothalamo–hypophyseal–gonadal axis level [10]. On the other hand, carbamates are also inhibitors of the cholinesterases in a similar way to organophosphates, but the inhibition induced by them is reversible in approximately 30 min [11]. In addition, pyrethroids are a group of synthetic insecticides derived from natural pyrethrins that have been used for more than 50 years and are included in 25% of the worldwide insecticide market [12]. The activity of pyrethroid insecticides consists of causing effects on sodium channels [13]. The principal target of pyrethroids is the sodium channels encoded by voltage-sensitive sodium channel gen codes [14,15]. Several studies have also proved that some pyrethroids can act as endocrine disruptors [16,17,18,19]. In this sense, some agencies, such as the International Agency for Research on Cancer, (IARC) have classified these pesticides according to their toxic effects; these include four organophosphates and the herbicide GLY, which is classified under the group 2A and 2B, “probably/possibly” cancer-causing agents, for instance, considering the limited evidence of carcinogenicity to humans (Table 2) [20]. In this regard, GLY, which is the most used herbicide in the world, is able to damage human DNA (deoxyribonucleid acid) and cause endocrine disruption, estrogen receptor and transcriptional activities inhibition, and cytotoxic effects which could be associated with GLY carcinogenetic effects [21,22].

**Figure 2:**
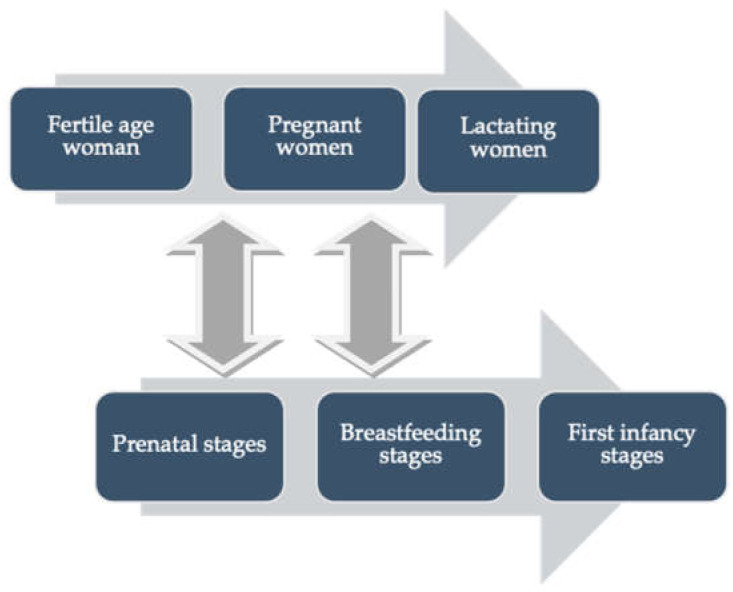
Stages of vulnerability related to the exposure to pesticides in women and children.

Although acute toxicity is well known for many pesticides, human data on their chronic effects are much more limited, even when chronic exposure to these xenobiotic compounds could trigger some biochemical alterations on target organs and lead to the appearance of several diseases [23]. Farmers represent a highly vulnerable population, due to the combination of unique social and cultural risk factors as well as exposure to hazards inherent in agricultural work [24]. Most of the research conducted to date has focused on operators or workers handling concentrated pesticides by loading, mixing, spraying, or cleaning equipment. However, pesticides can spread even very far distances from application areas via atmospheric transport, volatilization post-application, and spray drifts. Thus, it is important to determine the effects of long-term exposure to low levels of pesticide on residents surrounding intensive agricultural areas [1,24]. Therefore, when women are exposed over long periods of time to certain pesticides, several types of adverse reproductive outcomes may occur, leading to developmental problems in their offspring. Consequently, special attention should be given to these populations due to their vulnerability to pesticide exposure which might take place in different life stages (Figure 2) from preconception stages to infancy, while related diseases may manifest during infancy, adolescence or even adulthood [7,25].

### 3.2. Pesticides and Health Effects

Pesticide poisoning is a global public health concern, causing almost 300,000 deaths every year worldwide. Pesticide exposure is inevitable; there are four common ways pesticides can enter the human body: oral, dermal, via the eyes, and via the respiratory tract [26].

The most common biomarkers of pesticide exposure are AChE and butyrylcholinesterase (BChE) activity in both red blood cells and plasma. However, to determinate exposure to pesticides beyond organophosphates and carbamates, oxidative stress biomarkers are also used. Exposure to pesticides may not only increase the production of free radicals causing protein, nucleic acid, and lipid peroxidation, but also affect antioxidant capacity, including disturbances of the defense mechanisms. In this sense, Lukaszewicz-Hussain et al. [27] described the reactive species of oxygen (ROS) production by the conjugation between the organophosphate and cytochrome P450s, or by inhibiting the oxidative phosphorylation inducing a decrease in adenosine triphosphate (ATP) levels, which implicates an ROS production. In addition, Fareed et al. [8] confirmed that workers who presented inhibition in AChE and BChE also had alterations in parameters related to oxidative stress. All of these consequences have been linked to the development of diseases like cancer and renal and neurodegenerative diseases, together with reproductive health disorders [28,29].

Pesticide exposure among agricultural workers has been linked to certain types of cancer, DNA damage, oxidative stress, neurological disorders, and respiratory, metabolic, and thyroid effects [30]. Specifically, the chronic effects of organophosphates and carbamates have been correlated to weight loss, anemia, anorexia, impaired liver function, and delayed neuropathy [31]. The most reported chronic toxic effect caused by pesticides is carcinogenicity, being especially relevant the effects of insecticides in this feature [29,32]. Several studies have also demonstrated the effect of those pesticides as endocrine disruptors. Thus, organophosphates, carbamates, pyrethroids, and organochlorines can mimic the estrogenic function, acting as agonists and promoting a transcriptional activation of estrogen-responsive genes [29]. Particularly, Jin et al. [17] reported the estrogenic activity of cypermethrin and permethrin, similar to 17B-estradiol, in the MCF-7 human breast carcinoma cell line. Also, GLY-based herbicides presented endocrine disruption, inhibition in estrogen receptors and transcriptional activities [22]. Recently, Davoren et al. [21] concluded that the carcinogenic effects of GLY could be associated with its endocrine disruption activity.

### 3.3. Pesticides and Health Effects on Women, Pregnant and Lactating Women and Their Children

Many studies have linked occupational exposure to pesticides and problems in human male health primarily because the application of these pesticides is commonly performed by men. However, there is little information about the effects of these chemicals on the health of women both through occupational and non-occupational exposure (Table 3). Table 3 summarizes the two main types of exposure to pesticides in women, occupational and non-occupational, and the main forms of exposure that can occur.

The potential for occupational and environmental exposure to pesticides in women is important, and it can be even higher than for men in some situations, as there are a substantial number of women working in agriculture in many parts of the world. Thus, comprehensive data on the impact on women’s health are necessary (Table 4). In Table 4, the data found from different studies that have established some kind of health effect from exposure to different pesticides on different populations is shown. These health effects range from biochemical alterations and the presence of biomarkers for early renal damage in women to neurodevelopmental issues in their newborns. Several studies have linked these chemicals and their genotoxicity, immunotoxicity, and endocrine disruption effects as mechanisms that increase the risk of breast cancer in women [33,34]. Requena-Mullor et al. [23] established a relationship between gonadal diseases and high pesticide use in areas from the south of Spain in individuals who were diagnosed with ovarian and testicular cancer and dysfunction. The importance of occupational exposureis increasing due to the rise in the use of these chemicals to increase food production to address increasingly high demand [35]. This exposure on women of reproductive ages can also have consequences for their reproductive health, such as producing fertility disorders [36]. These consequences in reproductive health are reflected in characteristics like hormones and follicle count. In this sense, Jurewicza et al. [37] found a decrease in anti-Müllerian hormone (AMH) and follicle count and an increase in follicle-stimulating hormone (FSH) in women that presented 3-phenoxybenzoic acid (3-PBA), a pyrethroid metabolite, in urine. Related to reproductive health as well, Farr et al. [34] studied the effects of pesticides like carbamates or organophosphates in a population of applicators and spouses of applicators on farms in Iowa and North Carolina, establishing relationships between longer cycles of periods and missed periods and carbamates and herbicides exposure. These altered menstrual cycles can be related to the effects of endocrine disruption that most pesticides cause, resulting in reproductive system disturbances which can go from modulating the hormone concentrations, as previously established, to impaired fertility [28,38]. For pregnancy outcomes, pesticide exposures can be related to premature or spontaneous abortions [36]. Toxic effects have been reported not only in mothers but also in their children. In this regard, Chilipweli et al. [39] found that maternal pesticide exposure could potentially be associated with neurodevelopmental alterations and also related to factors such as distance from the farm, the area of residence of the pairs or the mother training in pesticide manipulation. Wielgomas and Piskunowicz [40] also showed that children from urban and rural areas of Poland had higher levels of pyrethroids than their parents, house dust and surface contamination being considered significant sources of exposure in children [41,42].

The impact of these health outcomes depends not only on the type of pesticide but also on the levels of these pesticides in the organism. These levels are modified by the characteristics of the type of exposure; the route of entry, non-occupational or occupational exposure, location, etc.

Some studies have been carried out to differentiate urban and rural exposures to pesticides to reach the non-occupational exposure sources for the general population. In this sense, Cruz et al. [44] detected higher levels of organochlorine residues in serum samples coming from the urban area of Coimbra in comparison to samples coming from rural areas of Portugal. Only hexachlorocyclohexane (HCH) isomer levels were higher for the rural population of Verride, with 16 of the 70 samples with values above the limit of quantification (LOQ) (Table 5). In Table 5, a classification of the data is compiled and proposed for the different studies depending on the type of analytical method used to detect diverse types of pesticides in various types of samples. The range of population, again, ranges from the general population including men and women to the more specific population of pregnant or breastfeeding women and their children. Likewise, inside these populations, a differentiation between rural and urban groups was made, and this was reflected in the obtaining of different levels of different pesticides in each group. Higher levels of diclorodiphenyltrichloroethane (DDT) have been linked to consumption of foods of animal origin, in addition todairy products because of the accumulation of fat, which is the reason for the detection of higher levels in urban populations that tend to consume imported products from countries still using DDT, while rural populations tend to consume the products they grow themselves [45]. In this regard, Wielgomas and Piskunowicz [40] measured levels of pyrethroids in urine samples, showing that in all the populations studied (none of them occupationally exposed), the levels of these pesticides were higher in rural areas of Poland than in urban ones. This exposure could come from products containing pyrethroids used for plant protection applied in the vicinity of the residential areas, especially for those living in agricultural areas. Children in both areas had higher concentrations of all metabolites than adults and, as Wielgomas and Piskunowicz [40] stated, this might be the evidence for additional exposure such as hand-to-mouth activity in children, house dust and surface contamination being considered a significant source of exposure to pesticides for infants and toddlers [42].

Pregnant women are a susceptible population to the potential hazards of pesticide exposure, not only for themselves but also for fetal exposure. Numerous studies have been carried out in pregnant women. For instance, Liu et al. [46], measured dialkylphosphate (DAP) levels in urine samples from a cohort of 450 pregnant women from the urban area of New York. They established that all DAP metabolites were detected in more than 80% of samples, with the exception of diethyldithiophosphate (DEDTP), which was only detected in 10% of the samples. Similar studies have been carried out in different countries, so in comparison to these levels of DAP, they were slightly higher than the ones from the Columbia Center for Children’s Environmental Health, another NYC-based cohort [47]. These levels were lower than for most European and Asian cohorts [48,49]. Many authors tried to associate the increase of these levels of DAP with vegetable and meat intake. In this sense, the Spanish INfancia y Medio Ambiente (Environment and Childhood, INMA, Barcelona, Spain) and the Canadian Maternal-Infant Research on Environmental Chemicals (MIREC, Ottawa, Canada) studies found an association between vegetable consumption and an increase in DAP levels [50,51]. Liu et al. [46] also found that meat was one of the main contributors to the increase of DAP levels. Xu et al., [52] found levels of 3-phenoxybenzoic acid (3PBA) in almost 90% of pregnant women from an urban area of China, and 2,2-dibromovinyl)-2,2-dimethylcyclopropane carboxylic acid (DBCA) was found in 72% of women and 4-fluoro-3-phenoxybenzoic acid (4F3PBA) was found in more than 95% of these women.

Lactation is also a very important concern because metabolites can reach children through breast milk. Sharma et al., [53] detected levels of both dichloro-diphenyl-dichloroethylene (DDE) and DDT in milk from breastfeeding mothers in different regions of the northwestern Himalayas in India. This may be due to the transformation of DDT into DDE, which is its more stable metabolite. Also, they found higher levels of DDT in primiparous mothers in comparison to multiparous women, and lower pesticide levels were detected in colostrum milk versus mature milk because of lower levels of fat in the colostrum. Also in India, but in the urban and semi-urban regions of Nadia and Kolkata, Anand et al., [54] carried out a study to detect organochlorines (OCs) pesticides and pyrethroids inthe breast milk of women. They detected levels of DDT and its metabolites, isomers of human chorionic gonadotropin (HCG), bifenthrin and endosulfan which were higher in the urban than in the semi-urban areas. In addition, they also detected levels of pyrethroids such as bifenthrin, permethrin and λ-cyhalothrin in the semi-urban areas. These differences in pesticide exposure have been linked to dietary habits, as previously stated above, and to the persistence of metabolites of pesticides in fat, therebefore linked specifically to the consumption of animal foods with high-fat content.

**Table 5:** Main pesticides detected in biological samples from women.

Population and Location of Study	Type and Characteristics of Sample	Type of Pesticides	Analythical Method	Levels of Pesticides Detected	Reference
Women and men from urban and rural populations in Coimbra (Portugal) *N* = 203 healthy volunteers, (*n* = 44) urban residents and (*n* = 159) rural residents all of them considered non-occupationally exposed	Serum separated from a 10 mL venipucture blood sample	OCs pesticides residues: HCH, Aldrin Dieldrin, HE, HCB, p,p’-DDT, o,p’-DDT, p,p’-DDE, p,p’- DDD, endosulfan sulphate	Liquid-liquid extraction with n-hexane-acetone (90 + 10). A clean up step with Florisil SPE cartridge Quantification with GC-ECD	The mean concentration level of all compounds analyzed was higher in urban samples except for HCH isomers. For HCH isomers, the mean total levels were higher in the Verride population (rural) with 13 +/- 36.6 μg/L. For p,p’DDE levels the higher level was 390.5 mg/L, in Coimbra but for the highest value found for o,p’DDT, it was in Ereira population with 256.7 mg/L. p,p’DDT was detected with the maximum value of 814.9 mg/L in the urban population. Both HCB and Endosulfan sulphate were detected in higher levels in Coimbra, with the maximum value of 393.3 mg/L and with 547.6 mg/L. Aldrin, dieldrin and HE were always below the LOQ in rural samples and for urban samples, these residues have been detected above the LOQ in a small number.	[44]
Women, non-pregnant nor lactating, from the European region of the Russian Arctic *N* = 204, divided into Nenets (*n* = 113) representing the indigenous population and non-Nenets (*n* = 91) consisting of mostly Russians permanently living in Nenets Autonomous Okrug (NAO)	Fasting blood sample	17 OCs pesticides: α-HCH,β-HCH, γ-HCH, p,p’-DDE, p,p’-DDD, o,p’-DDE, o,p’-DDD, heptachlor, cis-chlordane, trans-chlordane, cis-nonachlor, trans-nonachlor, aldrin, mirex, hexachlorobenzene, 1,2,3,5- tetrachlorobenzene, and 1,2,4,5-tetrachlorobenzene	Liquid-liquid extraction with H_2_SO_4_ and n-hexane. Quantification by GC-MS/MS triple quadrupole system	p,p′-DDE, o,p′-DDE, p,p′-DDD, HCB, β-HCH, aldrin, and mirex were detected in three samples and o o, p′-DDE, p,p′-DDE, HCB, and β-HCB, were found in 100% of the samples. The highest concentration was for p,p′-DDE: 68.8 ng/g lipid	[55]
Men and women and and their preschool- and school-aged children (or their children up to 18 years old) living in urban or rural areas in northern Poland *N* = 347 children and 190 adults. No farmers were included	Urine samples	Pyrethroid metabolites: 3-PBA, Br2CA, cis-Cl2CA and trans-Cl2CA	Extraction with hexane and analyzed by GC-MS	For Br2CA the detection rate was up to 27.4% in rural areas and was more than twice as high as in urban locations with a 12.6%. For cis-Cl2CA and trans-Cl2CA, in rural areas detection rates reached 56.6% and 61.9% while for urban areas, they were 39.1% and 42.5%, respectively	[40]
Pregnant women from New York (NYU CHES) (*n* = 450)	Urine samples	Metabolites of OP: DMP, DMTP DMDTP, DEP, DETP and DEDTP	Extraction with SPE cartridges.Quantification by HPLC-ESI-MS/MS	All DAP metabolites were detected in over 80% of the samples except for DEDTP, being median for DMP = 2.3 ng/mL and for DEP = 2.8 ng/mL, for DETP and DMTP and DMDTP values were lower (0.35 ng/mL, 1.8 ng/mL and 0.31 ng/mL)	[46]
Pregnant, healthy women on early pregnancy from a general hospital in Kunming City (China). *N* = 512 pregnant women recruited	Urine samples from one take during the early stages of pregnancy	Pyrethroids metabolites: 3-phenoxybenzoicacid (3PBA), 4-fluoro-3-phenoxybenzoic acid (4F3PBA), and 3-(2,2-dibromovinyl)-2,2-dimethylcyclopropane-1- carboxylic acid (DBCA)	Sample was extracted with ethyl acetate three times. The organic phase was gathered and evaporated to dryness using a rotary evaporator. PYR metabolites were detected using a sensitive ultra-performance liquid chromatography system (UPLC) coupled with a tandem mass spectrometry detector (MS/MS).	The detection frequency was 89.2% for 3PBA, 95.5% for 4F3PBA, and 72.9% for DBCA	[52]
Breastfeeding mothers from the north-western Himalayan region of India. *N* = 153	Breast milk samples	OCs (p,p’-DDT, p,p’-DDE) and chlorpyrifos	Extraction-cleanup through QuEChERS with a sample pretreatment with acetonitrile, anhydrous magnesium sulfate and anhydrous sodium chloride. Quantification by GC-MS	The concentrations of DDE that were found were higher than those for DDT in all maternal milk. In fact, the highest level of DDE detected was 0.064mg/kg milk.	[53]
Breastfeeding mothers from urban and semi-urban regions of the Nadia district and Kokalta (India). *N* = 81	Breast milk samples	OCs (HCHs, DDTs, endosulfan, aldrin and dieldrin) and pyrethroids (bifenthrin, λ-cyhalothrin and permethrin)	Liquid-liquid extraction with sulphuric acid/sodium chloride and ethyl acetate. Quantification by GC-MS	For metabolites that had higher values in urban areas than semi-urban ones, bifenthrin was detected with an average of 77 ng/g *w*/*w* for urban and 7 ng/g *w*/*w* for semi-urban, endosulfan which the average concentration of a and b-endosulfan were 12 ng/g *w*/*w* and 13 ng/g *w*/*w* respectively for the urban samples and 0.4 ng/g *w*/*w* and 2 ng/g *w*/*w* respectively for a and b in the semi-urban area. The sum of the average concentrations of DDT and its metabolites in the semi-urban and urban regions were 24 ng/g *w*/*w* and 294 ng/*w*/*w* respectively	[54]

OCs: organochlorines; HCH: hexachlorocyclohexane; HE: heptachlor epoxide; HCB: hexachlorobenzene; p,p’-DDT: 2,2, -bis (4-chlorophenil) -1,1,1-trichloroethane; o,p’-DDT: 2- (2-chlorophenil) -2 - (4-chlorophenil) -1,1,1-trichloroethane; p,p’-DDE: pp’diclorodifeniltricloroetane; p,p’- DDD: 1,1-dichloro-2,2-bis (p-chlorophenil) ethane; GC-ECD: Gas Chromatography on microcapillary column with an Electron Capture Detector source; LOQ: limit of quantification; α-HCH: isomer α- hexachlorocyclohexane; β-HCH: isomer β- hexachlorocyclohexane; γ-HCH: isomer γ- hexachlorocyclohexane; o,p’-DDE: 1-chloro-2-[2,2-dichloro-1-(4-chlorophenyl)ethenyl]-benzene; o,p’-DDD: [1,1-dichloro-2,2-bis(2,4=-dichlorophenyl)ethane; H_2_SO_4_: sulfuric acid; GC- MS/MS: gas chromatography coupled to a tandem mass spectrometry; PBA: bisphenol A; Br2CA: calcium bromide anhydrous; cis-Cl2CA: trans-3-(2,2-Dichlorovinyl)-2,2-dimethylcyclo-propane carboxylic acid, trans-Cl2CA: trans-3-(2,2-Dichlorovinyl)-2,2-dimethylcyclo-propane carboxylic acid, GC-MS: gas chromatography mass spectrometry; OP: organophospahate; DMP: dimethylpimelimidate; DMTP: Dimethyl Phosphorothioate; DMDTP: dimethyldithiophosphate; DEP: diethylphosphate; DETP: diethylthiophosphate; DEDTP: diethyl dithiophosphate; HPLC-ESI-MS/MS: high performance liquid chromatography- tandem mass spectrometry; QuEChERS: Quick, Easy, Cheap, Effective, Rugged & Safe.

## Figures and Tables

**Table 1:** Search in databases: different number of articles present in the two main data bases used for this review and included in the table that contains information relating pesticides and health effects in women and children.

Search Profile	Data Base	Number of Articles
Pesticides and health effects	SCOPUS	2983
Pesticides and health effects and women		223
Pesticides and health effects and children		432
Pesticides and health effects	PUBMED	8363
Pesticides and health effects and women		583
Pesticides and health effects and children		1217

**Table 2:** Classification of pesticides and examples.

Pesticide	Pest Target	International Agency for Research on Cancer Classification *	Target Organ
Organphosphates - Malathion - Diazinon - Tetrachlorvinphos - Parathion	Insecticide	2A 2A 2B 2B	Irreversible AChE ^+^ inhibitors
Carbamates - Carbofuran - Carbaryl	Insecticide		Reversible AChE inhibitors
Pyrethroids- λ-Cyhalothrin- Deltamethrin	Insecticide		Sodium channel modulators
GLY ^#^	Herbicide	2A	Aromatic Amino acid route in weeds

* 2A: probably carcinogenic to humans; 2B: possibly carcinogenic to humans; ^+^ AChE: Acetylcholinesterase; ^#^ GLY: glyphosate.

**Table 3:** Sources of exposure to pesticides in women.

Occupational Exposure	Non-Occupational Exposure
Application of agricultural pesticides	Residential areas bounded to plantations
Other agricultural tasks (re-entry in field for harvesting)	Residential areas where pesticides are sprayed (rice culture)
Farmers’ wives though home contamination	Food contamination (main route in general population)
Contamination of the workplace (structural treatments)	Pesticide contamination of water

**Table 4:** Studies of impact of pesticides exposure on womens’ health.

Location of Study	Population	Type of Pesticides	Health Effects	Reference
Southern Agricultural Growth Corridor of Tanzania community	Mothers and their youngest children from age 0–6 years Participants: *n* = 286, exposed: *n* = 172, unexposed/control: *n* = 114	Organophosphates: profenophos Pyrethroids: λ-cyhalothrin, chlorothalonil, imidaclopid and cypermethrin Carbamates: matalaxy + mancozeb and chlorpyrifos	Maternal pesticide exposure can be potentially associated with the neurodevelopment of their children, among the farmworker residents in comparison to the control. Other factors that can be significantly related are distance from the farm, the cluster of residence and advice and training on proper use and storage of pesticides, but also working and the duration of working during pregnancy can contribute to early neurodevelopment effects	[39]
Andalusia (Southern Spain): - Areas of high pesticide use: West Almeria, Centre of Almeria, South Granada, and Huelva Coastline - Areas of low pesticide use: Axarquia (Malaga), Jerez coastline (Cadiz), East Almeria, Northeast Jaen, North Cordoba, and North Seville	Men and women diagnosed with any gonadal disease (*n* = 5332): G1: high-exposure-area group: *n* = 2975 individuals G2: low-exposure-area group: *n* = 2357 Control group: 13,606 individuals without gonadal disease: G1: high-exposure-area group: *n* = 6647 individuals G2: low-exposure-area group: *n* = 6959	Macrocyclic lactones: abamectin and spinosad Neonicotinoids: imidacloprid, acetamiprid Pyrethroids: cypermethrin, deltamethrin and indoxacarb, azadirachtin, spiromesifen, Triazoles: tebuconazol, triadimenol, and miclobutanil, Anilino-pyrimidines: cyprodinil, mepanipyrim, pyrimethanil, Others: mepanipyrim, pyrimecolide, chlorthalonil, propamocarb, dimethomorph, azoxystrobin.	Four kinds of gonadal diseases were diagnosed using the International Classification of Diseases (ICD-9): ovarian cancer (183), testicular cancer (186), ovarian dysfunction (256), and testicular dysfunction (257). Average age of diagnosis in all these diseases was similar in both high pesticide use areas and low pesticide use areas. Ovarian and testicular cancer prevalence rates were significantly increased in areas of greater pesticide use in comparison with areas of lesser use. The risk for all diseases was also increased in these areas of higher pesticide use, the highest risk being observed for ovarian and testicular cancers	[23]
Marinaleda (Seville, Spain)	Women in fertile age (*n* = 39): *n* = 22 involved directly in the collection of fruits, *n* = 17 non-occupational exposure (NOE) group. The samples were collected in four periods every three months over a year	Pendimethalin, fluazifop-P-butyl, λ-Cyhalothrin, bromoxynil, GLY, dimethylamine, diflufenican, chlortoluron, tritosulfuron, imidacloprid, mancozeb, azoxystrobin and copper oxychloride	AChE activity was slightly lower in the women farmers group than in the NOE group in the last three periods of collection furthermore, women farmers had significantly higher levels of TBARS and carbonyl groups in the two first samplings, although in the last two samples ia decrease was observed in levels of TBARS and carbonyl groups in women farmers in comparison to the values of NOE. For biomarkers of early kidney damage, subclinical tubular damage is shown in women from rural settings that could progress to chronic kidney disease. These results suggest that the effect of pesticides could affect a general population in a rural environment based on agriculture and, therefore, possible preventive measures should be extended to the entire population	[3]
Canton Calvas, Province of Loja (Ecuador)	*N* = 115 women (18-65 years) from an agricultural-focused population: - Cariamanga (eminently commercial city, used as control group, *n* = 53), - Chimchanga (*n* = 29), - Colaisaca (*n* = 33)	Chimchanga farmers used: GLY, paraquat, propaquizafop and dichloro diphenyl trichloroethane (DDT) Colaisaca farmers used: these same pesticides and chlorpyrifos, dichlorvos, methamidophos and parathion, mepiquat chloride and cypermethrin	- Biochemical results: transaminases exhibited means higher than normal levels in the two exposed groups with respect to the control group. These values reveal a potential risk of liver injury after this long exposure to pesticides and their mixtures because these compounds are metabolized in the liver. - Genetic results and genotoxicity: for the Chimchanga females, death biomarkers karyolytic cells (KL) and karyorrhectic cells (KR) where higher than in the unexposed group. They stated that the exposure these women faced during the time of fumigation is causing liver and genetic damage in the population of Colaisaca due to the more frequent use of pesticides	[43]
Gdańsk, Poland	*N* = 511 women (25-39 years) with problems to achieve clinical pregnancy, but normal menstruation and confirmed ovulatory cycles without chronic diseases that may reduce ovarian reserve	Pyrethroid metabolites (cis-3- (2,2-dichlorovinyl)-2,2-dimethylcyclopropane carboxylic acid (CDDCA); trans-3-(2,2-dichlorovinyl)-2,2-di- methylcyclopropane carboxylic acid (TDDCA); 3-phenoxybenzoic acid (3PBA) and cis-2,2-dibromovinyl-2,2-di- methylcyclopropane-1-carboxylic acid (DBCA)	A relationship was found between 3-PBA concentrations and the decrease in the concentrations of anti-Mullerian hormones, and antral follicle count and increase in follicle-stimulating hormone levels	[37]
Iowa and North Carolina (USA)	Women (*n* = 3103) living on farms aged 21–40 years. These women were divided in three different groups according to: 1. Spouses of enrolled farmers, 2. Licensed female applicators, and 3. Those who were both spouses of enrolled farmers and licensed applicators themselves	Anilide, carbamate, dinitroaniline, organophosphate and triazine	Long period cycles were associated with mixing or applying any type of pesticide. These long cycles, in addition tomissed periods, were both related to increased days of pesticide use. The use of carbamates was specially associated with longer cycles, whereas use of herbicides was related to missed periods, just like the use of fumigants	[38]

G: group;

## References

[1] Doğanlar Z.B., Doğanlar O., Tozkir H., Gökalp F.D., Doğan A., Yamaç F., Aşkın O.O., Aktaş Ü.E. (2018). Nonoccupational Exposure of Agricultural Area Residents to Pesticides: Pesticide Accumulation and Evaluation of Genotoxicity. Arch. Environ. Contam. Toxicol..

[2] FAO Food and Agriculture Organization (2014). The International Code of Conduct on Pesticide Management.

[3] Martin-Reina J., Casanova A.G., Dahiri B., Fernández I., Fernández-palacín A., Juan D., Morales A.I., Moreno I. (2021). Adverse health effects in women farmers indirectly exposed to pesticides. Int. J. Environ. Res. Public Health.

[4] Venkidasamy B., Subramanian U., Samynathan R., Rajakumar G., Shariati M.A., Chung I.M., Thiruvengadam M. (2021). Organopesticides and fertility: Where does the link lead to?. Environ. Sci. Pollut. Res..

[5] European Food Safety Authority (2014). Guidance on the assessment of exposure of operators, workers, residents and bystanders in risk assessment for plant protection products. EFSA J..

[6] Md Meftaul I., Venkateswarlu K., Dharmarajan R., Annamalai P., Megharaj M. (2020). Pesticides in the urban environment: A potential threat that knocks at the door. Sci. Total Environ..

[7] Pascale A., Laborde A. (2020). Impact of pesticide exposure in childhood. Rev. Environ. Health.

[8] Fareed M., Kesavachandran C., Bihari V., Kamal R., Kuddus M. (2017). Oxidative stress and cholinesterase depression among farm workers occupationally exposed to pesticides in India. J. Environ. Biol..

[9] Ojha A., Yaduvanshi S.K., Srivastava N. (2011). Effect of combined exposure of commonly used organophosphate pesticides on lipid peroxidation and antioxidant enzymes in rat tissues. Pestic. Biochem. Physiol..

[10] Senthilkumaran B. (2015). Pesticide- and sex steroid analogue-induced endocrine disruption differentially targets hypothalamo-hypophyseal-gonadal system during gametogenesis in teleosts—A review. Gen. Comp. Endocrinol..

[11] Fukuto T.R. (1990). Mechanism of action of organophosphorus and carbamate insecticides. Environ. Health Perspect..

[12] Shafer T.J., Meyer D.A., Crofton K.M. (2005). Developmental neurotoxicity of pyrethroid insecticides: Critical review and future research needs. Environ. Health Perspect..

[13] Scott J.G. (2019). Life and death at the voltage-sensitive sodium channel: Evolution in response to insecticide use. Annu. Rev. Entomol..

[14] Soderlund D.M., Clark J.M., Sheets L.P., Mullin L.S., Piccirillo V.J., Sargent D., Stevens J.T., Weiner M.L. (2002). Mechanisms of pyrethroid neurotoxicity: Implications for cumulative risk assessment. Toxicology.

[15] Soderlund D.M. (2012). Molecular mechanisms of pyrethroid insecticide neurotoxicity: Recent advances. Arch. Toxicol..

[16] Garey J., Wolff M.S. (1998). Estrogenic and antiprogestagenic activities of pyrethroid insecticides. Biochem. Biophys. Res. Commun..

[17] Jin M., Li L., Xu C., Wen Y., Zhao M. (2010). Estrogenic activities of two synthetic pyrethroids and their metabolites. J. Environ. Sci..

[18] Ben Slima A., Chtourou Y., Barkallah M., Fetoui H., Boudawara T., Gdoura R. (2017). Endocrine disrupting potential and reproductive dysfunction in male mice exposed to deltamethrin. Hum. Exp. Toxicol..

[19] Singh D., Irani D., Bhagat S., Vanage G. (2020). Cypermethrin exposure during perinatal period affects fetal development and impairs reproductive functions of F1 female rats. Sci. Total Environ..

[20] (2015). World Health Organization IARC Monographs Volume 112: Evaluation of Five Organophosphate Insecticides and Herbicides. https://www.iarc.who.int/wp-content/uploads/2018/07/MonographVolume112-1.pdf.

[21] Davoren M.J., Schiestl R.H. (2018). Glyphosate-based herbicides and cancer risk: A post-IARC decision review of potential mechanisms, policy and avenues of research. Carcinogenesis.

[22] Gasnier C., Dumont C., Benachour N., Clair E., Chagnon M.C., Séralini G.E. (2009). Glyphosate-based herbicides are toxic and endocrine disruptors in human cell lines. Toxicology.

[23] Requena-Mullor M., Navarro-Mena A., Wei R., López-Guarnido O., Lozano-Paniagua D., Alarcon-Rodriguez R. (2021). Evaluation of gonadal alterations in a population environmentally exposed to a mixture of endocrine active pesticides. Int. J. Environ. Res. Public Health.

[24] Nassar P.P.M., Ribeiro M.G. (2020). Considerations for cholinesterase biomonitoring in flower and ornamental plant greenhouse workers. Sci. Total Environ..

[25] Frazier L.M. (2007). Reproductive disorders associated with pesticide exposure. J. Agromedicine.

[26] Alengebawy A., Abdelkhalek S.T., Qureshi S.R., Wang M.-Q. (2021). Heavy metals and pesticides toxicity in agricultural soil and plants: Ecological risks and human health implications. Toxics.

[27] Lukaszewicz-Hussain A. (2010). Role of oxidative stress in organophosphate insecticide toxicity—Short review. Pestic. Biochem. Physiol..

[28] Bretveld R.W., Thomas C.M.G., Scheepers P.T.J., Zielhuis G.A., Roeleveld N. (2006). Pesticide exposure: The hormonal function of the female reproductive system disrupted?. Reprod. Biol. Endocrinol..

[29] Mostafalou S., Abdollahi M. (2013). Pesticides and human chronic diseases: Evidences, mechanisms, and perspectives. Toxicol. Appl. Pharmacol..

[30] Curl C.L., Spivak M., Phinney R., Montrose L. (2020). Synthetic pesticides and health in vulnerable populations: Agricultural workers. Curr. Environ. Health Rep..

[31] Yushananta P., Ahyanti M., Anggraini Y. (2020). Risk of pesticides on anaemia events in horticulture farmers. Int. J. Innov. Creat. Chang..

[32] Mostafalou S., Abdollahi M. (2017). Pesticides: An update of human exposure and toxicity. Arch. Toxicol..

[33] Kim K.H., Kabir E., Jahan S.A. (2017). Exposure to pesticides and the associated human health effects. Sci. Total Environ..

[34] Teitelbaum S.L., Gammon M.D., Britton J.A., Neugut A.I., Levin B., Stellman S.D. (2007). Reported residential pesticide use and breast cancer risk on Long Island, New York. Am. J. Epidemiol..

[35] Silva Pinto B.G., Marques Soares T.K., Azevedo Linhares M., Castilhos Ghisi N. (2020). Occupational exposure to pesticides: Genetic danger to farmworkers and manufacturing workers—A meta-analytical review. Sci. Total Environ..

[36] Rani L., Thapa K., Kanojia N., Sharma N., Singh S., Grewal A.S., Srivastav A.L., Kaushal J. (2021). An extensive review on the consequences of chemical pesticides on human health and environment. J. Clean. Prod..

[37] Jurewicz J., Radwan P., Wielgomas B., Radwan M., Karwacka A., Kałużny P., Piskunowicz M., Dziewirska E., Hanke W. (2020). Exposure to pyrethroid pesticides and ovarian reserve. Environ. Int..

[38] Farr S.L., Cooper G.S., Cai J., Savitz D.A., Sandler D.P. (2004). Pesticide use and menstrual cycle characteristics among premenopausal women in the Agricultural Health Study. Am. J. Epidemiol..

[39] Chilipweli P.M., Ngowi A.V., Manji K. (2021). Maternal pesticide exposure and child neuro-development among smallholder tomato farmers in the southern corridor of Tanzania. BMC Public Health.

[40] Wielgomas B., Piskunowicz M. (2013). Biomonitoring of pyrethroid exposure among rural and urban populations in northern Poland. Chemosphere.

[41] Keenan J.J., Vega H., Krieger R.I. (2009). Potential exposure of children and adults to cypermethrin following use of indoor insecticide foggers. J. Environ. Sci. Health Part B Pestic. Food Contam. Agric. Wastes.

[42] Melnyk L.J., Byron M.Z., Brown G.G., Clayton C.A., Michael L.C. (2011). Pesticides on household surfaces may influence dietary intake of children. Environ. Sci. Technol..

[43] Arévalo-Jaramillo P., Idrobo A., Salcedo L., Cabrera A., Vintimilla A., Carrión M., Bailon-Moscoso N. (2019). Biochemical and genotoxic effects in women exposed to pesticides in Southern Ecuador. Environ. Sci. Pollut. Res..

[44] Cruz S., Lino C., Silveira M.I. (2003). Evaluation of organochlorine pesticide residues in human serum from an urban and two rural populations in Portugal. Sci. Total Environ..

[45] Covaci A., De Boer J., Ryan J.J., Voorspoels S., Schepens P. (2002). Distribution of organobrominated and organochlorinated contaminants in Belgian human adipose tissue. Environ. Res..

[46] Liu H., Campana A.M., Wang Y., Kannan K., Liu M., Zhu H., Mehta-Lee S., Brubaker S.G., Kahn L.G., Trasande L. (2021). Organophosphate pesticide exposure: Demographic and dietary predictors in an urban pregnancy cohort. Environ. Pollut..

[47] Engel S.M., Bradman A., Wolff M.S., Rauh V.A., Harley K.G., Yang J.H., Hoepner L.A., Barr D.B., Yolton K., Vedar M.G. (2016). Prenatal organophosphorus pesticide exposure and child neurodevelopment at 24 months: An analysis of four birth cohorts. Environ. Health Perspect..

[48] Van den Dries M.A., Pronk A., Guxens M., Spaan S., Voortman T., Jaddoe V.W., Jusko T.A., Longnecker M.P., Tiemeier H. (2018). Determinants of organophosphate pesticide exposure in pregnant women: A population-based cohort study in the Netherlands. Int. J. Hyg. Environ. Health.

[49] Hioki K., Ito Y., Oya N., Nakayama S.F., Isobe T., Ebara T., Shibata K., Nishikawa N., Nakai K., Kamida T. (2019). Intra-individual variations of organophosphate pesticide metabolite concentrations in repeatedly collected urine samples from pregnant women in Japan. Environ. Health Prev. Med..

[50] Llop S., Murcia M., Iñiguez C., Roca M., González L., Yusà V., Rebagliato M., Ballester F. (2017). Distributions and determinants of urinary biomarkers of organophosphate pesticide exposure in a prospective Spanish birth cohort study. Environ. Health A Glob. Access Sci. Source.

[51] Sokoloff K., Fraser W., Arbuckle T.E., Fisher M., Gaudreau E., LeBlanc A., Morisset A.S., Bouchard M.F. (2016). Determinants of urinary concentrations of dialkyl phosphates among pregnant women in Canada—Results from the MIREC study. Environ. Int..

[52] Xu Q., Zhu B., Dong X., Li S., Song X., Xiao X., Zhang C., Lv Y., Zhang X., Li Y. (2020). Pyrethroid pesticide exposure during early pregnancy and birth outcomes in southwest china: A birth cohort study. J. Toxicol. Sci..

[53] Sharma N., Chandel R., Sharma I., Sharma P., Gurung B. (2020). Pesticides contamination of lactating mother’s milk in the north-western Himalayan region of India. J. Environ. Biol..

[54] Anand N., Chakraborty P., Ray S. (2021). Human exposure to organochlorine, pyrethroid and neonicotinoid pesticides: Comparison between urban and semi-urban regions of India. Environ. Pollut..

[55] Varakina Y., Lahmanov D., Aksenov A., Trofimova A., Korobitsyna R., Belova N., Sobolev N., Kotsur D., Sorokina T., Grjibovski A.M. (2021). Concentrations of persistent organic pollutants in women’s serum in the European arctic Russia. Toxics.

